# Chromosome and Plasmids of the Tick-Borne Relapsing Fever Agent *Borrelia hermsii*

**DOI:** 10.1128/genomeA.00528-16

**Published:** 2016-06-09

**Authors:** Alan G. Barbour

**Affiliations:** Departments of Microbiology & Molecular Genetics, Medicine, and Ecology & Evolutionary Biology, University of California Irvine, Irvine, California, USA

## Abstract

The zoonotic pathogen *Borrelia hermsii* bears its multiple paralogous genes for variable antigens on several linear plasmids. Application of combined long-read and short-read next-generation sequencing provided complete sequences for antigen-encoding plasmids as well as other linear and circular plasmids and the linear chromosome of the genome.

## GENOME ANNOUNCEMENT

*Borrelia hermsii* exists in nature among rodents and their argasid tick ectoparasites in mountainous regions of western North America (1). Besides its importance as a zoonotic human pathogen, *B. hermsii* is renowned for its multiphasic system of antigenic variation for immune evasion (2). The several antigens constituting its diverse repertoire are encoded by genes on different linear plasmids of 25 to 60 kb (3, 4). While the megabase linear chromosomes of 5 strains of *B. hermsii* are publicly available, sequences of their plasmids, which make up 30 to 40% of the genome, have remained incomplete and largely in unassembled fragments. The many paralogous sequences distributed among different replicons present a challenge for plasmid assembly (5).

The single-molecule real-time (SMRT) long-read approach (6) on a PacBio RS I instrument (Pacific Biosciences, Menlo Park, CA) was combined with the short paired-end read approach on an Illumina (Hayward, CA) HiSeq 2500 instrument, as described in reference 7, for sequencing the chromosome, linear megaplasmid, linear plasmids, and circular plasmids of the “Browne Mountain” isolate of type strain HS1 (BioProject PRJNA311246 and BioSample SAMN04481062) of *B. hermsii*. DNA extracted with the Qiagen (Valencia, CA) Midi kit from cells grown in BSK II medium (3) was sheared to 20 to 50 kb for library preparation. The 98,902 reads from two SMRT cells had an *N*_50_ read length of 20,536 nucleotides (nt), provided an average coverage of 854×, and were assembled with Hierarchical Genome Assembly Process 2 of SMRT Analysis v2.3 (Pacific Biosciences). Illumina reads of 70 to 200 nt numbered 107,636,008, provided coverage of >1,000×, and were assembled *de novo* with CLC Assembly Cell v8.5 (Qiagen, Denmark). Prediction of protein-coding sequences and annotation of the chromosome and megaplasmid were performed by the Prokaryotic Genome Annotation Pipeline v3.1 (http://www.ncbi.nlm.nih.gov/genome/annotation_prok/), followed by manual annotation. Other sequences were manually annotated.

The chromosome sequence comprises 922,500 bp with a G+C content of 29.8%. Alignment of this sequence with that of a geographically separate source of strain HS1, isolate DAH (accession no. CP000048; BioProject PRJNA29637), identified 45 (0.005%) single nucleotide polymorphisms (SNPs) (34 transitions and 11 transversions), 5 indels of ≤6 nt, and 2 copy number variants distinguishing them. Through fuller accounting of internal direct repeats with long-read sequencing, the HS1 megaplasmid sequence was revised upwards to 182,541 bp from the previous estimate of 173,739 bp (8). The sizes of plasmid lpE27, which bears the primary expression site (9), and plasmids lpN31 and lpF27, which bear the archived versions of antigen genes *vlpA7* and *vlpA21*, respectively, corresponded to their physical measurements by pulsed-field gel electrophoresis (4). Three additional plasmids—lpB58, which carries the essential *resT* telomere resolvase gene for *Borrelia* spp. (10), lpV47, and lpF27—were also found to carry archival sequences for the *vsp* and *vlp* genes for antigenic variation.

### Nucleotide sequence accession numbers.

The sequences for the chromosome, megaplasmid, and plasmids cp28, lpB58, lpE27, lpN31, lpT28, lpV47, lpG27, cp6.5, lpF27, and pR have been deposited in the GenBank/DDBJ/EMBL database under accession numbers CP014349, CP014350, CP014351, CP014792, CP014871, CP015331, CP015332, CP015333, CP015334, CP015335, CP015336, and CP015337, respectively.
